# Identifying the psychological effects of nocebo education: results from two pre-registered experiments

**DOI:** 10.1007/s10865-024-00520-3

**Published:** 2024-09-21

**Authors:** Kim J. Görner, Emily K. Spotts, Andrew L. Geers

**Affiliations:** Department of Psychology, University of Toledo, Toledo, OH 43606 USA

**Keywords:** Side effects, Nocebo, Placebo, Patient education, Pain, Expectation

## Abstract

Providing treatment side effect information to patients increases the risk of harm due to the nocebo effect. Nocebo education, in which patients learn about nocebo effects, is a novel strategy that can be used across a variety of situations and individuals to decrease unpleasant treatment side effects. It is currently unclear which psychological changes are induced by nocebo education, which is information required to maximize this intervention. Two pre-registered studies investigated the effects of nocebo education on side effect expectations, side effect control beliefs, feelings toward treatments, intentions to avoid or seek side effect information, and perceptions of treatment efficacy. In Study 1 (*N* = 220), adult participants either watched or did not watch a nocebo education intervention video prior to reading vignettes about receiving a surgical treatment for pain and a medication for pain. Study 2 (*N* = 252) was similar to Study 1, with the inclusion of a health behavior video control group and participants only reading about a medication treatment for pain. In both experiments, nocebo education reduced global side effect expectations and increased side effect self-efficacy beliefs. Nocebo education also increased intentions to avoid side effect information and decreased intentions to seek more side effect information. Evidence was inconclusive on whether nocebo education changes affective associations with the treatments. The findings demonstrate that nocebo education has a multi-faceted influence with the potential to change patient behavior. The results can be used to improve the management of adverse treatment side effects.

**Supplementary Information:**

The online version contains supplementary material available at 10.1007/s10865-024-00520-3.

Disclosing the potential adverse side effects of medical treatments allows patients to make informed decisions regarding their care options, which aligns with the ethical principle of patient autonomy. Unfortunately, experimental and clinical research finds that the mere knowledge of treatment side effects increases the likelihood of side effects, a phenomenon referred to as the nocebo effect (Colloca & Miller, 2011). A meta-analysis of 130 experiments with various nocebo inductions found the magnitude of the nocebo effect across a range of outcomes, including pain, nausea, and exercise-related responses, was medium in size, *g* = 0.52 (Rooney et al., 2024). Nocebo effects are unpleasant experiences and may lead to adverse downstream consequences including increased burden of illness, reduced quality of life, and discontinuation or avoidance of medically indicated treatments (Bingel et al., 2011; Faasse, 2019; Mitsikostas et al., 2012; Pan et al., 2019; Rezk & Pieper, 2017). In sum, side effect information increases patient autonomy but also increases the risk for nocebo effects.

It may be possible to devise ethically permissible interventions that minimize nocebo effects. One such technique is nocebo education (Barsky et al., 2002; Crichton & Petrie, 2015; MacKrill et al., 2021; Pan et al., 2019). Nocebo education refers to informing patients about the nature and possible impacts of the nocebo effect (Quidde et al., 2018). This often takes the form of describing the nocebo effect and symptom misattribution in lay terms using everyday examples to illustrate these effects (e.g., MacKrill et al., 2021). Recent research provides evidence that nocebo education can decrease side effects. For example, participants reported fewer symptoms from sham windfarm-generated infrasound following nocebo education, as compared to participants not receiving this education (Crichton & Petrie, 2015). Also, in a sample of patients undergoing chemotherapy, nocebo education led to a reduction in the intensity of adverse events reported two weeks later (Michnevich et al., 2022).

A benefit of nocebo education is that it could be used in a variety of contexts, with ill and non-ill individuals, for vaccinations or medical treatment, and with established patients or first visits to a primary care provider. Nocebo education interventions are now being advocated for reducing side effects across a host of medical domains (Colloca, 2024; Evers et al., 2018; Geers et al., 2022; Kleine-Borgmann & Bingel, 2018; Manaï et al., 2019). Although nocebo education may be an effective strategy for reducing nocebo effects, the psychological mechanisms by which the intervention alters side effect experiences remain poorly understood. In addition to impacting side effect experience, they may also influence other feelings, cognitions, and actions. Therefore, the present research investigates the psychological changes caused by nocebo education. The goal is to identify candidate mediators that can be targeted to increase the effectiveness of nocebo education on side effect reduction. Further, by measuring a range of psychological outcomes, we hope to clarify the wider impact of nocebo education.

## Potential mechanisms of nocebo education

### Side effect expectations

Substantial research suggests that nocebo effects, like placebo effects, are caused by expectations (Manaï et al., 2019; Mondloch et al., 2001; Petrie & Rief, 2019). Expectations are not the only driving forces behind nocebo effects, however, as anxiety, attention, and associative learning also contribute to nocebo effects (Faasse, 2019; Geers et al., 2021). Nevertheless, extensive data supports the position that expectations are a key cause of nocebo effects (Colloca, 2024; Webster et al., 2016).

Given the established role of expectations in causing nocebo effects, nocebo education may lessen nocebo effects by reducing the side effect expectations (Petrie & Rief, 2019). Although a viable explanation, currently there is little evidence supporting the position that nocebo education changes side effect expectations (Meijers et al., 2022). Therefore, we tested if expectations differed between individuals who were and were not provided nocebo education. To provide a strong test of this hypothesis, we assessed side effect expectations in several ways: measuring global and specific expectations and assessing expectations for side effects that were and were not disclosed for a treatment.

### Side effect control beliefs

Nocebo education may evoke psychological changes other than side effect expectations. For example, in nocebo education, individuals are informed that treatment side effects are not only the result of the pharmacological properties of active treatments but also result from psychological processes, such as expectations and learning. This knowledge may foster a sense of empowerment and a belief that treatment side effects are under a patient’s personal control (Barsky, 2017; Michnevich et al., 2022), which has the potential to alter side effect experiences. Such an outcome would be consistent with research showing that control perceptions can lessen negative experiences, such as pain and discomfort (Rokke et al., 2004; Rokke & Lall, 1992). Further, studies find that giving participants personal control over their treatment through choice-making reduces nocebo effects (Bartley et al., 2016; Faasse et al., 2023). Consequently, nocebo education may increase control beliefs and thereby reduce nocebo effects.

### Affective associations with the treatment

Another possibility is that nocebo education changes feelings toward a treatment. That is, knowing side effects are not immutable consequences of a treatment could reduce anxiety and raise feelings of hope. Reduced anxiety and raised hope can shape perceptions and diminish negative feelings such as pain and distress (Basten-Günther et al., 2019; Snyder et al., 2005). Further, inducing positive affect can dampen nocebo effects, whereas increasing distress can strengthen nocebo effects (Geers et al., 2019; Roderigo et al., 2017). Consequently, rather than working through a cognitive pathway, nocebo education may alter side effect experience through an affective pathway.

### Side effect information seeking and avoiding intentions

It is also possible that nocebo education influences side effect-related behaviors that, in turn, modify side effect experience. Relevant to this idea, Nestoriuc et al. (2021) found that individuals receiving nocebo education desired less side effect information about antidepressant medications than those not receiving this information. These findings suggest that in addition to directly shaping side effect experience, nocebo education could indirectly change experience by influencing the side effect information one obtains. Here, we followed up on the results of Nestoriuc et al. (2021) to determine if nocebo education changes behavioral intentions to seek or avoid side effect information.

## Present research

Two pre-registered experiments investigated candidate mediators through which nocebo education reduces side effects. The experiments examined the influence of nocebo education on four psychological factors: side effect expectations, side effect control beliefs, feelings toward treatments, and intentions to avoid or seek side effect information. Based on the aforementioned research, we hypothesized that nocebo education: (1) lowers expectations of side effects, (2) increases feelings of control about managing side effects, (3) reduces negative and increases positive affect toward treatments, and (4) increases behavioral intentions to avoid side effect information and lowers intentions to seek side effect information. Finally, in Study 2 we assessed if nocebo education changes beliefs in treatment’s effectiveness and in the nocebo effect.

## Study 1

In Study 1, nocebo education was manipulated and afterwards participants were asked to imagine themselves in two treatment vignettes. Potential side effects were provided for both treatments. After reading each vignette, participants completed the dependent measures.

## Methods

### Participants and design

Community adults (*N* = 220) were recruited from Prolific, an online survey recruitment system (Palan & Schitter, 2018). The sample size was determined by a power analysis conducted with G*Power (Faul et al., 2009). Based on the effect size from pilot data (*f* = 0.3), and α = 0.05 (two-tailed), the analysis estimated a minimum sample size of 208. Sample ages ranged from 20 to 76 (*M*_*age*_ = 42.21, *SD* = 13.76), 49.1% identified as male, 48.2% identified as female, and 2.8% identified as another gender or preferred not to disclose gender (see Table 1 for further demographic information). Participants lived in the United States at the time of study, were fluent in English, and were compensated for their participation. The study was approved by the University Institutional Review Board and the procedure and analysis plan were pre-registered on the Open Science Framework (OSF; osf.io/szh7g).

This study utilized a between-within-subjects design. After providing informed consent, participants were randomly assigned to one of two between-subject conditions: receiving nocebo education (experimental condition) or not receiving nocebo education (control condition). For the within-subject variable, all participants read two fictional medical vignettes that mentioned possible side effects.

**Table 1:** Participant characteristics for Studies 1 and 2

	Study 1 (*N* = 220)				Study 2 (*N* = 252)			
	*M*	*SD*	*N*	%	*M*	*SD*	*N*	%
Age	42.21	13.76			42.92	14.12		
Gender								
Male			108	49.1			121	48.0
Female			106	48.2			119	47.2
Non-binary			4	1.8			8	3.2
Self-identified another gender			1	0.5			2	0.8
Declined to answer			1	0.5			2	0.8
Racial background								
White			176	80.0			209	82.9
Black/African American			20	9.1			19	7.5
American Indian/Alaska Native			4	1.8			2	0.8
East Asian			10	4.5			11	4.4
South Asian			2	0.9			4	1.6
Native Hawaiian/Pacific Islander			1	0.5			0	0
Another race			11	5.0			3	1.2
Declined to answer			3	1.4			5	2.0
Health Status								
Poor			10	4.5			9	3.6
Fair			39	17.7			34	13.5
Good			89	40.5			88	34.9
Very good			66	30.0			98	38.9
Excellent			16	7.3			23	9.1
Regular medication intake			141	64.1			154	61.1
Health care system contact								
Once a year or less			101	45.9			112	44.4
2–5 times a year			80	36.4			98	38.9
6–11 times a year			23	10.5			26	10.3
About once a month			10	4.5			13	5.2
More than once a month			6	2.7			3	1.2

### Procedures

After providing informed consent and answering preliminary questions, participants were randomly assigned to the nocebo education or control condition. The nocebo education was presented in a six-minute video about the negative effects of the nocebo effect, which was modeled closely on the nocebo education intervention developed by Critchon and Petrie (2015; also see De Brochowski et al., 2023; MacKrill et al., 2021). The script for this video is located in the supplemental materials. The video provided participants with a general definition of the nocebo effect, followed by a discussion of commonly experienced bodily symptoms. Three examples were used to illustrate the role of attention in the experience of common bodily symptoms and their misattribution as side effects. Finally, the video reviewed scientific findings that suggest lowered expectations of side effects may reduce side effects. Those in the control condition did not see this video.

Following the nocebo education manipulation, participants read two randomly ordered medical vignettes, one involving the treatment of back pain with a fictional medication (labeled Relaxodol) and the other involving the treatment of back pain with a surgical intervention. In the vignettes, participants imagined they were experiencing back pain, and the treatment (medication or surgery) was prescribed by their doctor. Each treatment was described as having potential side effects. The vignettes included one of two synonymous lists of potential side effects of the treatment, such as drowsiness or fatigue, and nausea or upset stomach (see Supplemental Table 2). The two side effect lists were counterbalanced between the two vignettes to avoid confounding side effects and medical vignettes. Participants then completed the dependent measures and responded to demographic questions.

### Measures

#### Global side effect expectations

We included two measures of side effect expectations, one global and one specific. Global side effect expectations were assessed with five items, including, “To what extent do you expect side effects from undergoing the surgery?”. Two of the items were on 4-point scales and three were on 7-point scales. Since items were on different scales, we used Aiken’s (1987; Glick et al., 2004) formula for scale conversion to equate the ratings on the 1 to 4 scale and then the items were averaged to form a global side effect expectation scale for each vignette (medication scale, α = 0.80; surgery scale, α = 0.84).

#### Specific side effect expectations

To assess specific side effect expectations, we used twelve items derived from the General Assessment of Side Effects Scale (GASE; Rief et al., 2011; von Blanckenburg et al., 2013). Items included the six side effects mentioned in the medical vignettes (prompted side effects) as well as six additional side effects that were not mentioned (unprompted side effects). Each symptom was rated on a Likert-type scale ranging from 0 (*not expected*) to 3 (*severe*). Items were summed to create prompted and unprompted side effect scales for each vignette.

#### Side effect self-efficacy beliefs

Perceptions of the ability to control side effects were assessed using five items on a 7-point Likert scale ranging from 1 (*strongly disagree*) to 7 (*strongly agree*). A sample item is “I do not have to experience side effects when taking [treatment] if I don’t want to.” The items were reverse scored when necessary and averaged for each of the vignettes. Both scales displayed good internal consistency (medication scale, α = 0.90; surgery scale, α = 0.92).

#### Affective associations with the treatment

The emotions associated with the treatments were measured using a 15-item scale. Nine items inquired about positive emotions, such as “I am optimistic about my reaction to the [treatment]”, and six items inquired about negative emotions, such as “I am scared I will have a bad reaction to the [treatment]”. Responses were provided on 7-point Likert scales. Preliminary analyses found that the positive and negative items were highly intercorrelated. As such, the negative items were reverse scored and averaged with the positive items to create a treatment affective association scale for each of the vignettes (medication scale, α = 0.95; surgery scale, α = 0.95).

#### Side effect information seeking and avoiding intentions

Behavioral intention estimates were assessed with six items regarding seeking side effect information and six items regarding avoiding side effect information. Participants received the prompt “When receiving treatments in the future, how likely are you to:” with individual items such as “talk to others (e.g., friends, family) about their experiences with the treatment?” and “avoid reading the side effect information as much as possible?” Responses to the 12 items were made on a slider scale ranging from 0 (*extremely unlikely*) to 100 (*extremely likely*). Preliminary analyses indicated the seeking and avoiding items were only moderately related. As such, separate seeking and avoiding scales were created for each vignette by averaging the respective items (medication treatment avoiding: α = 0.91; medication treatment seeking: α = 0.84; surgery treatment avoiding: α = 0.90; surgery treatment seeking: α = 0.83).

### Statistical analysis

It was hypothesized that receiving nocebo education would increase positive responses (e.g., expect fewer side effects, feel more self-efficacious) as compared to not receiving nocebo education (control condition). This hypothesis was anticipated to occur on dependent measures related to both the medical and surgery vignettes. To test these predictions, scores on the dependent measures were submitted to separate 2 (condition) by 2 (treatment type) between-within subject ANOVAs.

It should be noted that the presented analyses diverged in two ways from the registration plan. These deviations were documented and posted in the Open Science Framework project page (osf.io/szh7g). First, we anticipated including a single-item measure to assess belief in the nocebo effect following the condition manipulation. This measure was inadvertently omitted and thus nocebo beliefs were not examined in Study 1. This measure was, however, included in Study 2. Second, the plan was to test our hypotheses using independent t-tests. That plan, however, neglects the within-subject factor and does not allow for an assessment of whether any effects of condition differed based on treatment type. As such, we instead conducted 2 × 2 between-within ANOVAs to provide these direct comparisons. The results of the independent t-tests do not diverge from the ANOVA tests, and they are provided in Supplemental Table 1. All analyses were performed using SPSS 28.0 (IBM Corp, 2021).

## Results

Means and standard deviations on all Study 1 measures are in Table 2.

**Table 2:** Means and standard deviations of all dependent measures for Study 1

	Nocebo Education (*n* = 109)		Control (*n* = 111)	
	M	SD	M	SD
Global SE expectations ^a^	3.57	0.93	3.99	0.92
Global SE expectations ^b^	4.29	0.96	4.59	0.90
Prompted SE expectations ^a^	2.36	2.01	3.03	2.13
Prompted SE expectations ^b^	3.55	2.40	3.86	2.36
Unprompted SE expectations ^a^	1.50	1.77	1.97	1.92
Unprompted SE expectations ^b^	2.24	2.28	2.49	2.03
SE self-efficacy beliefs ^a^	3.57	1.22	2.92	1.20
SE self-efficacy beliefs ^b^	3.19	1.28	2.68	1.13
Treatment affective associations ^a^	5.18	1.15	4.73	1.14
Treatment affective associations ^b^	4.60	1.27	4.24	1.19
SE information seeking ^a^	43.92	22.74	53.57	22.29
SE information seeking ^b^	48.19	21.20	58.14	22.82
SE information avoiding ^a^	53.09	25.13	34.25	23.93
SE information avoiding ^b^	53.16	24.51	34.95	22.65

*Note* SE = side effect; ^a^ medication treatment; ^b^ surgery treatment

### Global side effect expectations

The 2 × 2 ANOVA on global side effect expectations yielded a significant main effect of condition, *F*(1, 218) = 11.22, *p* < .001, *η*_*p*_^*2*^ = 0.05. Nocebo education participants were less likely to expect side effects (*M* = 3.92) than control participants (*M* = 4.29). This analysis also produced a large main effect of treatment type, *F*(1, 218) = 108.07, *p* < .001, *η*_*p*_^*2*^ = 0.33, indicating participants anticipated more side effects from surgery (*M* = 4.44) than the medication (*M* = 3.78). The ANOVA indicated that the Condition × Treatment Type interaction was not significant, *F*(1, 218) = 0.92, *p* = .34, *η*_*p*_^*2*^ < 0.01.

### Specific side effect expectations

The 2 × 2 ANOVA on prompted side effects produced no main effect of condition, *F*(1, 217) = 3.53, *p* = .06, *η*_*p*_^*2*^ = 0.02. An examination of the means shows a non-significant trend for the nocebo education participants to expect fewer prompted side effects (*M* = 2.95) than control participants (*M* = 3.45). The treatment type main effect was significant, *F*(1, 217) = 45.97, *p* < .001, *η*_*p*_^*2*^ = 0.18. Participants expected more of the prompted side effects from surgery (*M* = 3.70) than the medication (*M* = 2.70). The Condition × Treatment Type interaction was not significant, *F*(1, 217) = 1.47, *p* = .23, *η*_*p*_^*2*^ = 0.01.

Analysis of unprompted side effects did not produce a significant effect of condition, *F*(1, 217) < 2.38, *p* = .13, *η*_*p*_^*2*^ = 0.01. The treatment type main effect was significant, *F*(1, 219) = 19.63, *p* < .001, *η*_*p*_^*2*^ = 0.08, indicating participants expected more unprompted side effects from the surgery (*M* = 2.23) than the medication (*M* = 1.87). The interaction was not significant, *F*(1, 217) < 0.61, *p* = .44, *η*_*p*_^*2*^ < 0.01.

### Side effect self-efficacy beliefs

When beliefs about the ability to control side effects were examined, we found a significant main effect of condition, *F*(1, 218) = 15.18, *p* < .001, *η*_*p*_^*2*^ = 0.07. Participants in the nocebo education condition expressed stronger beliefs in their ability to control side effects (*M* = 3.38) than controls (*M* = 2.80). There was also a main effect of treatment type, such that participants reported higher self-efficacy beliefs in responses to the medication (*M* = 3.25) than to the surgery (*M* = 2.94), *F*(1, 218) = 20.04, *p* < .001, *η*_*p*_^*2*^ = 0.08. These main effects were not qualified by an interaction, *F*(1, 218) = 1.0, *p* = .32, *η*_*p*_^*2*^ = 0.01.

### Affective associations with the treatment

Analysis of the affective association measure yielded a main effect of condition, *F*(1, 218) = 8.45, *p* < .005, *η*_*p*_^*2*^ = 0.04, as well as a main effect of treatment type, *F*(1, 218) = 45.30, *p* < .001, *η*_*p*_^*2*^ = 0.17. Nocebo education participants expressed more positive associations with the treatments (*M* = 4.89) than control participants (*M* = 4.49), and participants held more positive associations with the medication treatment (*M* = 4.96) than the surgery treatment (*M* = 4.42). The condition by treatment interaction was not significant, *F*(1, 218) = 0.35, *p* = .55, *η*_*p*_^*2*^ < 0.01.

### Side effect information seeking and avoiding intentions

A 2 × 2 ANOVA on side effect information seeking resulted in a main effect of condition, *F*(1, 218) = 12.40, *p* = .001, *η*_*p*_^*2*^ = 0.06, revealing that those receiving nocebo education expected to seek less side effect information (*M* = 45.86) than control participants (*M* = 56.30). There was also a treatment type main effect, *F*(1, 218) = 18.52, *p* < .001, *η*_*p*_^*2*^ = 0.09, showing that participants anticipated seeking out more information on the side effects of surgery (*M* = 53.17) than the medication (*M* = 49.00). The Condition × Treatment Type interaction was not significant, *F*(1, 218) = 0.25, *p* = .62, *η*_*p*_^*2*^ < 0.01.

Finally, we submitted the avoiding side effect scale to a 2 × 2 ANOVA. This analysis yielded a main effect of condition, *F*(1, 218) = 36.09, *p* < .001, *η*_*p*_^*2*^ = 0.15, indicating those receiving nocebo education believed they would avoid side effect information (*M* = 53.68) more than control participants (*M* = 34.20). There was no main effect of treatment type, *F*(1, 218) < 0.10, *p* = .80, *η*_*p*_^*2*^ < 0.01, nor a significant interaction, *F*(1, 218) < 1.98, *p* = .16, *η*_*p*_^*2*^ = 0.01.

## Study 1 discussion

Study 1 found that nocebo education reduces expectations for treatment side effects. This effect was significant for global ratings, but not for specific symptom ratings. Nocebo education also produced other psychological changes. Specifically, compared to controls, those given nocebo education had a stronger belief that they could control their side effects, expected to have more positive responses to treatments, expected to seek out less side effect information, and expected to be more avoidant of side effect information. The results show nocebo education does not just alter side effect expectations but that the intervention has a multi-faceted influence. For example, the findings suggest that nocebo education could change patient behavior by reducing how much side effect information they subsequently acquire about a treatment. Notably, although reactions were more negative to the surgery than the medication treatment, the effect of nocebo education was equivalent across the two vignettes.

## Study 2

Study 2 was conducted to partially replicate and extend the findings of Study 1. A key change was the inclusion of a second control condition. In Study 1, nocebo education participants watched the education video, whereas controls did not watch any video. Thus, the two conditions diverged in the receiving of an intervention as well as the content of that intervention. To provide a tighter control, we added a control condition in which participants watched a health-related video that was designed to be similar in style, but different in content, to the nocebo education video. Second, and related to this change, we assessed beliefs in the nocebo effect before and after the manipulation to verify nocebo education was successful in changing this belief. Participants in the two video conditions also evaluated the quality of the video they viewed. This measure was included to verify that the nocebo education video was not perceived as superior to the control video, which would hinder the interpretation of Study 2.

The constructs assessed in Study 1 were again assessed in Study 2. Two additional dependent measures were added. First, we included a measure to assess the desire to avoid side effect information. This measure captures a general motive to avoid side effects, not simply the belief in changing actions in reference to the specific medical treatment provided. Second, we added in a measure assessing perceptions of treatment efficacy. To our knowledge, no study has examined whether nocebo education changes perceptions of treatment effectiveness.

Finally, we simplified the design of Study 2 and used only the medication vignette from Study 1.

## Methods

### Participants and design

Participants were community adults (*N* = 252) recruited through Prolific. A minimum sample size of *N* = 225 was determined using G*Power (Faul et al., 2009), based on averaging the effects from Study 1, with power = 0.90, and α = 0.05 (two-tailed). Ages ranged from 20 to 76 (*M*_*age*_ = 42.21, *SD* = 13.76), 49.1% identified as male, 48.2% identified as female, and 2.8% identified as another gender or preferring not to disclose gender (see Table 1 for sample characteristics). All participants lived in the United States at the time of the study and were fluent in English. Participants were compensated for their participation. Study approval was obtained by the University ethics board and the procedures and data analysis plan were pre-registered on the OSF (osf.io/827hx).

This study employed a between-subjects design, with participants randomly assigned to one of three conditions: nocebo education video, control (health behavior) video, and no video.

### Procedures

All participants provided informed consent at the start of the study. The procedures were similar to Study 1, with the nocebo education participants viewing the same video administered in Study 1. The control video was a four-and-a-half-minute educational video about health behaviors, which was modeled closely after the nocebo education video. The video discussed common health risks of modern sedentary lifestyles and insufficient, irregular sleep. Following this, suggestions on how one can address these health concerns were offered. Finally, the video summarized scientific evidence that suggests potential increases in daily activity and a more regular sleep schedule can prevent negative health impacts.

### Measures

#### Belief in the nocebo effect

Belief in the nocebo effect was assessed with a single item: “Receiving information about side effects can increase the actual occurrence of side effects”. Responses were made on a scale ranging from 1 (*strongly disagree*) to 7 (*strongly agree*). The item was completed at the start of the study and again after the video manipulation.

#### Evaluation of the stimulus videos

Four questions, derived from prior research (MacKrill et al., 2021), were used to assess quality evaluations (e.g., “Did the video make sense?”). Responses ranged from 1 (*not at all*) to 7 (*completely*). These four items were averaged and used to assess if the videos were perceived similarly (α = 0.84).

#### Global side effect expectations

Four items were included to assess global side effect expectations, such as “To what extent do you expect side effects from using Relaxodol?”. The items were slightly altered versions of those from Study 1. In Study 2, all items were measured on a 7-point Likert scale and were averaged (α = 0.82).

#### Specific side effect expectations

To measure specific side effect expectations, we provided participants with a list of six side effects prompted in the medical vignette they read (e.g., fatigue, nausea) and six not in the vignette (e.g., headache, difficulty urinating). Participants selected the side effects they expected and the number selected was summed to create prompted and unprompted side effect expectation scales, each ranging from 0 to 6.

#### Desire to avoid side effect information

A scale was included to assess one’s global desire to avoid side effect information. This scale (Clemens et al., 2024) was developed based on the Information Avoidance Scale (Howell & Shepperd, 2016). An example item is, “When it comes to side effects of medications, sometimes ignorance is bliss.” It is an eight-item measure, with responses provided on a 7-point Likert scale ranging from 1 (*strongly disagree)* to 7 (*strongly agree*). All eight items were averaged to create a summary scale (α = 0.93).

#### Expectations for treatment efficacy

Six items from the multi-faceted Treatment Expectation Questionnaire (TEX-Q; Shedden-Mora et al., 2023) were added to assess expected treatment efficacy. The six items selected were those assessing treatment benefits and positive treatment impact (items 1 to 6). All responses were provided on a 7-point Likert scale. A sample item is “How much do you expect Relaxodol will improve your quality of life?”. Responses were averaged to create an expectation for treatment efficacy scale (α = 0.93).

#### Other measures

The measures to assess side effect self-efficacy beliefs (α = 0.89), affective associations with the treatment (α = 0.95), side effect information seeking and avoiding intentions (seeking: α = 0.82; avoiding: α = 0.86), and demographics were the same as those used in Study 1.

### Statistical analysis

To assess the effectiveness of the nocebo education manipulation, scores on both the pre- and post-belief in nocebo effect item were submitted to a repeated-measures ANOVA, with nocebo education condition represented as a 3-level between-subject variable. Following the ANOVA, two planned comparisons were conducted. The first tested the hypothesis that the increase in belief in nocebo effects would be larger in the nocebo education condition vs. the two control conditions (1 vs. 2 contrast comparison). The second planned comparison tested if nocebo belief scores differed between the two control conditions. Next, the video evaluation scores were submitted to an independent t-test, to determine if the nocebo education and control videos were evaluated similarly.

The primary hypothesis was that nocebo education would produce more positive responses (e.g., expect fewer side effects, feel more self-efficacious) as compared to the two control conditions. To examine this hypothesis, scores on the dependent measures were submitted to separate one-way ANOVAs, with condition represented as a 3-level independent variable. To test our specific predictions, two planned comparisons were conducted. The first tested if responses were more positive (e.g., expected fewer side effects) in the nocebo education condition as compared to the two control conditions (1 vs. 2 contrast comparison). The second comparison tested if scores differed between the two control conditions (0, -1, 1 contrast comparison). All analyses were performed using SPSS 28.0 (IBM Corp, 2021).

## Results

Means and standard deviations on the Study 2 measures are presented in Table 3.

**Table 3:** Means and standard deviations of all dependent measures for Study 2

	Nocebo education (*n* = 83)		Video control (*n* = 84)		Control (*n* = 85)	
	M	SD	M	SD	M	SD
Nocebo belief pre-score	4.59	1.17	4.40	1.30	4.56	1.47
Nocebo belief post-score	5.39	1.23	4.14	1.48	4.28	1.55
Video Evaluations	7.40	0.72	7.51	0.68		
Global SE expectations	3.57	1.13	3.99	1.04	3.84	1.12
Prompted SE expectations	2.48	1.71	3.01	1.67	2.74	1.75
Unprompted SE expectations	0.64	0.88	0.65	0.87	0.86	1.06
SE self-efficacy beliefs	3.55	1.22	3.09	1.28	3.34	1.17
Treatment affective associations	5.09	1.19	4.89	1.26	4.94	1.18
Desire for SE information	3.07	1.39	2.38	1.30	2.37	1.26
SE information seeking	50.01	22.38	59.38	23.19	59.77	20.13
SE information avoiding	52.37	24.83	36.57	24.42	39.61	23.21
Treatment efficacy expectations	7.48	1.55	7.44	1.80	7.29	1.88

*Note* SE = side effect

### Belief in the nocebo effect

A 2 × 3 repeated measures ANOVA on beliefs in the nocebo effect produced a condition main effect, *F*(2, 249) = 7.49, *p* = .001, *η*_*p*_^*2*^ = 0.06, and the anticipated Measure × Condition interaction, *F*(2, 249) = 25.73, *p* < .001, *η*_*p*_^*2*^ = 0.17. Planned contrasts confirmed that beliefs in the nocebo effect increased more for the nocebo education participants (*M*_*change*_ = 0.80) as compared to the video control (*M*_*change*_ = − 0.26) and no video control (*M*_*change*_ = − 0.28) participants, *t*(249) = 7.16, *p* < .001, *d* = 0.91. The control conditions did not differ, *t*(249) = 0.12, *p* = .91, *d* = 0.02.

### Evaluation of the stimulus videos

When scores on the video evaluation measure were entered into an independent t-test, the analysis yielded no significant effect, *t*(165) = 0.99, *p* = .32, *d* = 0.15. That is, the nocebo education video (*M* = 7.40) and the control video (*M* = 7.51) were not evaluated differently.

### Global side effect expectations

The one-way ANOVA on the global side effect measure produced a marginal effect of condition, *F*(2, 249) = 3.14, *p* = .05, *η*_*p*_^*2*^ = 0.03. The first planned comparison indicated that participants receiving nocebo education had lower expectations for side effects (*M* = 3.57) than those in the video (*M* = 3.99) and no video (*M* = 3.84) control conditions, *t*(249) = 2.36, *p* < .05, *d* = 0.30. The two control conditions did not differ, *t*(249) = 0.89, *p* = .38, *d* = 0.11.

### Specific side effect expectations

Contrary to prediction, neither the ANOVA for the prompted, *F*(2, 249) = 2.10, *p* = .14, *η*_*p*_^*2*^ = 0.02, nor the unprompted side effect measures, *F*(2, 249) = 1.44, *p* = .24, *η*_*p*_^*2*^ = 0.01, produced significant effects. Neither of the planned comparisons were significant.

### Side effect self-efficacy beliefs

The one-way ANOVA testing side effect control beliefs yielded a marginally significant effect, *F*(1, 249) = 2.98, *p* = .05, *η*_*p*_^*2*^ = 0.02. Planned comparisons found that those in the nocebo education condition held a stronger belief in their ability to control side effects (*M* = 3.55) than the video (*M* = 3.09) and the no video controls (*M* = 3.34), *t*(249) = 2.05, *p* < .05, *d* = 0.26. The two control conditions did not differ, *t*(249) = 1.34, *p* = .18, *d* = 0.17.

### Affective associations with the treatment

Inconsistent with our prediction and Study 1, the affective association scores did not differ by condition, *F*(1, 249) = 0.61, *p* = .55, *η*_*p*_^*2*^ = 0.01. The two planned comparisons were not significant, *p*s > 0.28.

### Desire to avoid side effect information

We tested if the desire for side effect information differed by condition. The one-way ANOVA produced a significant effect of condition, *F*(2, 249) = 7.72, *p* = .001, *η*_*p*_^*2*^ = 0.06. Planned comparisons indicated that those receiving nocebo education had a greater desire to avoid side effect information (*M* = 3.07) than participants in the video (*M* = 2.38) and no video (*M* = 2.37) control conditions, *t*(249) = 3.92, *p* < .001, *d* = 0.50, with the two control conditions not differing, *t*(249) = 0.03, *p* = .97, *d* < 0.01.

### Side effect information seeking and avoiding intentions

The one-way ANOVA testing beliefs about seeking side effect information resulted in a significant main effect, *F*(1, 249) = 5.31, *p <* .01, *η*_*p*_^*2*^ = 0.04. Planned comparisons found that participants in the nocebo education condition expected to seek less side effect information (*M* = 50.01) than participants in the video control (*M* = 59.38) and the no video control (*M* = 59.77) conditions, *t*(249) = 3.25, *p* = .001, *d* = 0.41. The two control conditions did not differ, *t*(249) = 0.12, *p* = .91, *d* = 0.02.

Similarly, avoiding side effect information scores also yielded a significant effect in a one-way ANOVA, *F*(1, 249) = 10.04, *p* < .001, *η*_*p*_^*2*^ = 0.08. Participants receiving nocebo education expected they would avoid side effect information more (*M* = 52.37) than participants in the video control (*M* = 36.57) and no video control (*M* = 39.61) conditions, *t*(249) = 4.41, *p* = .001, *d* = 0.56. The two control conditions did not differ, *t*(249) = 0.82, *p* = .41, *d* = 0.10.

### Expectations for treatment efficacy

Finally, we tested if the nocebo education manipulation altered expectations for treatment effectiveness. The one-way ANOVA did not produce a significant effect, *F*(1, 249) < 0.28, *p* = .76, *η*_*p*_^*2*^ < 0.01, and neither planned contrast was significant, *p*s > 0.55. Thus, nocebo education did not change expectations for treatment efficacy.

## Study 2 discussion

Study 2 was a pre-registered partial replication of Study 1 with an additional control condition. The results found no difference between the no video and video control conditions. Further, the nocebo education and health (control) videos received similar quality evaluations. The results also indicated that the nocebo education intervention successfully changed beliefs in the nocebo effect, as compared to controls. Taken together, these findings support the internal validity of the present paradigm.

In terms of the primary outcomes, Study 2 aligned with Study 1 in finding that those given nocebo education expected fewer side effects on a global side effect expectation measure, but not on a specific side effect expectation measure. Also, following nocebo education, participants again reported a stronger belief that they could control their side effects, expected to seek out less side effect information, and expected to be more avoidant of side effect information. In contrast to Study 1, participants did not expect to have more positive responses to the treatment. Also, Study 2 found that nocebo education did not alter perceptions of overall treatment effectiveness.

## General discussion

Two pre-registered experiments with adult U.S. community samples examined the immediate psychological changes that follow nocebo education. In both studies, nocebo education changed global side effect expectations, side effect self-efficacy, and behavioral intentions towards acquiring side effect information. These results were found in response to both a medical and a surgical treatment vignette. Nocebo education modulated affective treatment associations in one study but did not significantly alter specific side effect expectations in either study. In sum, the experiments identify three reliable psychological pathways by which nocebo education may diminish side effects.

Across both studies, global expectations for side effects were reduced by nocebo education. This finding aligns with prior theorizing that nocebo effects work by modifying negative expectations (Mondloch et al., 2001; Petrie & Rief, 2019). While nocebo education consistently diminished global side effect expectations, changes in specific prompted or unprompted side effects were weaker in magnitude and non-significant in both studies. Notably, prior nocebo education studies have focused primarily on specific prompted and unprompted side effect expectations (De Brochowski et al., 2023; Michnevich et al., 2022). In those studies, nocebo education generated little change on expectation scales. Taken together, these results suggest that nocebo education has a stronger influence on generalized rather than specific beliefs about side effects.

If global expectations are influenced more by nocebo education than specific expectations, it raises several questions. For example, why might this result emerge? One possibility is that nocebo education involves explaining general psychological processes causing side effects, including expectations, misattribution, and conditioning. These global explanations may more easily shape global expectations than specific expectations. This possibility is congruent with conceptualizations, such as construal level theory (Trope & Liberman, 2010), that describe cognitive processing as varying from high (abstract) to low (concrete) levels. In this line of research, generalized information and instructions are more apt to change global rather than specific mental structures. Although speculative, that might explain the divergence in the global and specific side effect results found here. A second question that arises concerns the relative role of global and specific expectations for patient responses. Whereas some conceptualizations point to the critical role of specific expectations in guiding responses (Kirsch, 1999), others focus on both global and specific expectations (Carver & Scheier, 1998). One possibility is that global expectations influence general impressions of symptoms, whereas specific side effect expectations influence individual symptom appraisals. If correct, it may be possible to design education interventions to target both global and specific expectations to maximize beneficial outcomes.

Both studies found that nocebo education increases one’s self-efficacy for managing side effects. These results suggest that explaining side effects as a consequence of psychological and contextual factors provides a greater sense of control over them. It should be noted that two recent studies have also tested whether nocebo education changes expectations of side effect control. In one of these studies (Meijers et al., 2022), nocebo education increased control expectations, primarily when combined with an empathy induction. The second study did not find this effect (Michnevich et al., 2022). It is notable that both of these prior studies examined the influence of nocebo education on perceptions of chemotherapy side effects. There may be specific aspects about chemotherapy that make changing a patient’s feelings of control particularly challenging, such as the widely known adverse side effects (e.g., loss of hair). Alternatively, it may be that the non-clinical samples, as used in the present research, respond to nocebo education with greater feelings of control. If this is the case, it suggests an advantage of early use of nocebo education interventions, such as at patient intake visits, as it may raise control perceptions.

A third class of measures altered by nocebo education concern side effect information desires and intentions. First, conceptually replicating the results of Nestoriuc et al. (2021), Study 2 found that nocebo education reduces the desire for side effect information. Further, in both studies participants estimated that they would seek out less side effect information and also actively avoid this information. Coupled together, the findings point to an indirect pathway by which nocebo education might changes nocebo effects: by changing avoidance and seeking behaviors. As side effect information exposure arrives from a wide array of sources, such as social media, news reports, family, and friends (Clemens et al., 2023; Tan et al., 2022), these behavior changes could diminish nocebo effects in patients. Importantly, however, our studies only measured behavioral intentions. Although intentions are often a predictor of actual behavior, the two do not necessarily align (Webb & Sheeran, 2006). Future research should assess if information seeking and avoiding behaviors are modulated by nocebo education.

The finding that nocebo education changes intentions to seek and avoid side effect information raises the possibility that nocebo education can alter many behaviors. For example, as expectations about potential side effects have been linked to lower levels of treatment adherence (Menckeberg et al., 2008; Pietrzykowski et al., 2022), it is possible that nocebo education improves treatment adherence by changing side effect expectations. Similarly, self-efficacy and personal control are instrumental in increasing behavioral intentions as well as the amount of effort one is willing to expend to complete a behavior (Bandura, 1982; Maddux et al., 1982). Because nocebo education increases side effect control expectations, it may, in turn, improve patients’ willingness and effort to engage in and adhere to treatments with adverse side effects. Future research should assess these possibilities.

Another contributing factor important to treatment adherence and outcomes is positive feelings toward treatments (Baloush-Kleinman et al., 2011; Beck et al., 2011; Mohamed et al., 2009). While nocebo education led to increased positive and decreased negative affect towards treatments in Study 1, these findings were not replicated in Study 2. As it is unclear why this difference emerged, the role of affective treatment evaluations in nocebo education warrants further investigation.

Finally, Study 2 tested if nocebo education modifies treatment efficacy expectations. This is notable, as efficacy expectancies are a driver of treatment responses (Haanstra et al., 2015; Petrie & Rief, 2019). Although side effect expectations can be associated with treatment efficacy perceptions (Heisig et al., 2016), we found nocebo education did not affect treatment efficacy expectations.

### Clinical implications

Taken together, the results of previous studies and the present research suggest that implementing nocebo education into clinical practice can offer many benefits, such as increased feelings of control and reduced side effects, without interfering with treatment efficacy beliefs. The usefulness of nocebo education for clinical practice was previously highlighted by an interdisciplinary expert consensus group on placebo and nocebo effects (Evers et al., 2021). In the expert consensus, however, it was noted that clinicians must be careful so that conversations about nocebo effects do not increase negative affect (e.g., anxiety) or raise the belief that side effects are likely or out of one’s personal control. Such outcomes could detract from the benefits of nocebo education. The current results are notable, as we found that educating individuals about nocebo effects did not increase negative feelings. Further, nocebo education reduced global side effect expectations and raised feelings of control. Given these results, the nocebo education intervention employed here may reduce nocebo effects and avoid the potential concerns raised by the expert consensus group. The current nocebo education presentation (see supplemental materials) provided a general definition of the nocebo effect, followed by an explanation of its causes, several everyday examples, and concluded with recommendations on how to avoid nocebo side effects. If clinicians discuss nocebo effects with patients, a similar approach may help avoid unwanted outcomes that could arise when describing nocebo effects.

It should be noted that, in addition to nocebo education, other strategies have been proposed for curbing nocebo effects. One such strategy is positively framing side effect information, which can involve placing emphasis on the majority of patients not experiencing a side effect rather than on the minority that do (Barnes et al., 2019; Mao et al., 2021). Another nocebo reduction approach involves shared decision-making, in which the clinician asks the patient if they would prefer to not be informed about all possible treatment side effects (Colloca, 2017; Geers et al., 2023). If the patient does not wish to receive full disclosure, then the clinician and patient could collaboratively determine what side effect information is to be disclosed. With multiple nocebo reduction methods available, clinicians are able to weigh the available options and select a strategy that best fits a particular treatment context and a patient’s characteristics. Ultimately, the greatest benefit to patients may come from combining nocebo education with the other approaches. For example, in an initial visit, a clinician could provide nocebo education to a patient so that they are aware of this general phenomenon. Later, when explaining the side effects of a specific treatment, positive framing could be employed.

### Strengths and limitations

The presented research has both strengths and limitations. Strengths include the use of two well-powered pre-registered experiments, with two control conditions, that tested predictions across both medical and surgery vignettes. Additionally, we assessed multiple candidate mediators of nocebo education that were selected based on prior research. In terms of limitations, the studies were conducted with online non-clinical samples rather than within clinical settings and populations. Although nocebo education can be used with general populations, it will be informative to examine this intervention with a variety of patient samples. Further, the medical vignettes were fictional and limited to pain treatment, so it is not yet known if our findings generalize beyond the selected study material. Future research should move from these “proof of concept” experiments into clinical settings. That said, vignette and video-based methodologies are useful in clarifying basic processes of medical interventions before the involvement of patient samples (Meijers et al., 2022; Peerdeman et al., 2021; Van Vliet et al., 2013). Finally, the present studies use a single nocebo education intervention. Although the intervention was derived from prior studies (Critchon & Petrie 2015; MacKrill et al., 2021), there are various approaches that could be used and research should compare types of content and formats (e.g., verbal, leaflets, video).

## Conclusions

In two experiments, nocebo education changed global side effect expectations, side effect self-efficacy, and behavioral intentions towards side effect information. The findings suggest nocebo education may have the ability to influence many medically relevant outcome variables, including patient behaviors. Nocebo education interventions could be strategically designed to further maximize these psychological changes to strengthen effectiveness. The results of this study may aid the development of optimal materials to inform patients of nocebo effects and thereby reduce adverse patient outcomes.

## Electronic supplementary material

Below is the link to the electronic supplementary material.

Supplementary Material 1

## Data Availability

The deidentified data underlying the results presented in this study may be made available upon request from the corresponding author Dr. Andrew L. Geers.

## Code availability

Statistical analysis was performed on SPSS version 28.
