# Supplementary material for: Identifying the psychological effects of nocebo education: results from two pre-registered experiments
Source: J Behav Med. 2024 Sep 21;47(6):1080–93. doi: 10.1007/s10865-024-00520-3 (PMC11499334; doi:10.1007/s10865-024-00520-3)
Supplement: Supplementary file 1 — Supplementary Material 1 [file 10865_2024_520_MOESM1_ESM.docx]

***Supplemental Table 1.* Results of preregistered independent samples t-tests for Study 1.**

|  | Nocebo Education  (*n* = 109) | | Controls  (*n* = 111) | | *t* | *p* | Cohen’s *d* |
| --- | --- | --- | --- | --- | --- | --- | --- |
|  | *M* | *SD* | *M* | *SD* |  |  |  |
| Global SE expectations ^a^ | 3.57 | 0.93 | 3.99 | 0.92 | 3.33 | .001 | .45 |
| Global SE expectations ^b^ | 4.29 | 0.96 | 4.59 | 0.90 | 2.43 | .016 | .33 |
| Prompted SE expectations ^a^ | 2.36 | 2.01 | 3.03 | 2.13 | 2.15 | .033 | .29 |
| Prompted SE expectations ^b^ | 3.55 | 2.40 | 3.86 | 2.36 | 0.97 | .331 | .13 |
| Unprompted SE expectations ^a^ | 1.50 | 1.77 | 1.97 | 1.92 | 1.90 | .059 | .26 |
| Unprompted SE expectations ^b^ | 2.24 | 2.28 | 2.49 | 2.03 | 0.85 | .397 | .12 |
| SE self-efficacy beliefs ^a^ | 3.57 | 1.22 | 2.92 | 1.20 | -3.94 | <.001 | -.53 |
| SE self-efficacy beliefs ^b^ | 3.19 | 1.28 | 2.68 | 1.13 | -3.10 | .002 | -.42 |
| Treatment affective associations ^a^ | 5.18 | 1.15 | 4.73 | 1.14 | -2.93 | .004 | -.40 |
| Treatment affective associations ^b^ | 4.60 | 1.27 | 4.24 | 1.19 | -2.16 | .032 | -.29 |
| SE information seeking ^a^ | 43.92 | 22.74 | 53.57 | 22.29 | 3.18 | .002 | .43 |
| SE information seeking ^b^ | 48.19 | 21.20 | 58.14 | 22.82 | 3.20 | .002 | .45 |
| SE information avoiding ^a^ | 53.09 | 25.13 | 34.25 | 23.93 | -5.70 | <.001 | -.77 |
| SE information avoiding ^b^ | 53.16 | 24.51 | 34.95 | 22.65 | -5.46 | <.001 | -.77 |

*Note*. SE = side effect.

^a^ medication treatment
^b^ surgery treatment

**Nocebo Education Video Script (Studies 1 and 2)**

I would like to talk to you today about how we experience side effects and symptoms, and some of the ways our mind works to either make us pay more, or less, attention to the physical sensations in our bodies. I would also like to describe something to you called the nocebo effect, which is a psychological phenomenon which explains why some people may be more inclined than others to experience side effects, or physical symptoms, after they take a medication or receive a medical treatment.

In medicine, studies have found that in the general population many people will experience some kind of physical symptom during the course of their daily lives. The experience of physical sensations or symptoms is actually very common, with around half of the population reporting five or less symptoms per week. Many of these symptoms may be described as general symptoms - non-specific - such as fatigue, drowsiness, upset stomach, or nausea. Experiencing these general symptoms will not ordinarily affect peoples’ daily activities. Rather, the daily experience of some type of bodily sensation or physical symptom is an ordinary part of the normal human experience.

Awareness of these common bodily sensations and physical symptoms vary and people will pay attention to them only if asked about them or if something draws their attention to the symptoms. Attention is drawn to symptoms and sensations when we take new medications. At these times, people often want to see what kind of effect the medication has on them. This greater attention can lead people to misinterpret their generic symptoms as signs of an adverse health event or illness, which may not be the case. Thus, one of the main reasons for people experiencing more than the normal amount of symptoms from a medication is that they are simply paying greater attention to bodily sensations after taking the medication. At these times, people can attribute the cause of their general physical symptoms and sensations to the medication they are taking. Another reason may be that people’s expectations or beliefs about the probability of experiencing symptoms may make them misattribute normal bodily sensations as symptoms. Our brain and body are highly connected, and some studies have shown that the expectation of experiencing symptoms can actually result in the appearance of those symptoms, even if there is no apparent reason for this to happen.

The process of buying a new car serves as a useful example of the attentional focus towards symptoms that I just mentioned. Say you have been looking to purchase a new car, and after test driving a few you decide to buy a car you had not previously considered, a VW Golf, and you wish to buy a red colored one. You decide also to take a week or two to think about it. What you may find is that quite quickly during those two weeks you will start to notice Red VW Golf cars when in traffic, or out walking. You will notice more and more of those cars. You will even start to be more aware of VW Golfs that are not red in color. That is because due to your intention to purchase one, your attention has been focused towards cars of this model. You expect to own one soon. This attentional focus is often operating without your complete awareness, but as soon as you see a VW Golf you think of the car you wish to buy. Studies have shown that these attentional processes operate in a variety of situations. For example, while watching a film in a cinema, if one person coughs often several people may also cough. This is because our urge to cough may be suppressed while our attention is held by the movie, but when an individual in the audience coughs, our awareness of this person’s behavior turns our attention rapidly to our own urge to cough, and so we cough, as do others in the audience. A further example is when a group of people are out for a meal in a restaurant. We have a strong expectation that eating food can positively or negatively affect the way we physically feel before, during, or after a meal. If one person at the table mentions they are feeling some discomfort or dissatisfaction from the meal, in many cases someone else at the table will start to pay more attention to how they are feeling while eating, and may themselves start to feel similar, where before hearing of the other persons’ discomfort they did not notice anything unusual in themselves.

When taking a medication or drug treatment, while there are many positive benefits, there is also the possibility of a negative experience in terms of physical symptoms. In medicine we commonly refer to these adverse symptoms as side effects. The nocebo effect, which I mentioned earlier, is a term used to describe the psychological process whereby our expectations of experiencing side effects, alongside our general beliefs about medications, can influence whether or not we actually experience any of the side effects of a medication we are taking. Taking into account the attentional processes I just talked about, if we combine our expectation with being overly attentive, then while taking a medication we may regularly scan our bodies for any sign of physical sensations or side effects. In this way we may heighten our chances of attributing normal physical sensations as side effects and feeling more unwell. Conversely, if our expectation is low that we will experience side effects or symptoms, there is a reasonably good chance that we will not notice any of those symptoms and have a more positive experience while taking the medication.

This is important to remember when receiving side effect information from a health professional. Studies show that even mentioning the possibility of irritable or annoying side effects may cause us to experience these symptoms. This has even happened in studies where no drug was taken at all by the participants – they have actually taken a placebo, or inactive, medication. This is evidence of our expectations of annoying symptoms sometimes creating our actual experience, as I just mentioned. Often, few people will actually experience these annoying symptoms, and this is especially so if they have not been told about the possibility of having those symptoms. So, research suggests it will be helpful if you can think about ways to lower or remove any expectation that you will have side effects, and also if you can keep yourself optimistic about the positive outcomes taking the medication will have for you. In this way you can protect yourself against experiencing the discomfort of side effects, and hopefully have a better physical outcome while taking your medication.

**Treatment Vignettes for Study 1 and Study 2**

**Fictional Treatment Vignette - Medication**

Imagine the following:

When helping your friend move into a new house, you hurt yourself trying to lift a heavy couch. Since then, you have been experiencing lower back pain. To find out what is causing your pain, you see a doctor. After the doctor examines your back, he informs you that you strained your muscles in your lower back. To relieve you from your pain and help your muscles to heal, your doctor prescribes you the muscle relaxant, Relaxodol. This medication works through reducing the tension in your muscles and relieving pain.

Your doctor then goes on to inform you about the potential side effects of the medication. Relaxodol may lead to *fatigue, nausea, high body temperature, difficulty or inability to sleep, hallucinations*, and *shortness of breath*.

**Fictional Treatment Vignette - Surgery**

Imagine the following:

You have been experiencing increasing pain in your lower back for almost two months. You have tried using heat treatments, stretching exercises, and resting – all without success. To find out what is causing your pain, you see a doctor. After the doctor examines your back, he informs you that you have a herniated disc. Spinal discs are rubbery cushions that sit between your spinal bones. When a disc is herniated, the soft center of the spinal disc is pushed through a crack in the tougher exterior casing, which can lead to the pain you have been experiencing. To relieve you from your pain, your doctor informs you that you will need to undergo surgery. He recommends Microdiscectomy Spine Surgery. During the procedure, the excess disc material will be removed.

Your doctor then goes on to inform you about the potential side effects of the surgery. The surgery may lead to *fatigue, nausea, high body temperature, difficulty or inability to sleep, hallucinations, and shortness of breath*.

*Supplemental Table 2.* Synonymous side effect lists for Study 1.

| **List 1** | **List 2** |
| --- | --- |
| Fatigue | Drowsiness |
| Nausea | Upset stomach |
| High body temperature | Fever |
| Difficulty of inability to sleep | Insomnia |
| Hallucinations | Mental disorientation |
| Shortness of breath | Difficulty breathing |
